# Morphoproteomics, E6/E7 in-situ hybridization, and biomedical analytics define the etiopathogenesis of HPV-associated oropharyngeal carcinoma and provide targeted therapeutic options

**DOI:** 10.1186/s40463-017-0230-2

**Published:** 2017-08-17

**Authors:** Robert E. Brown, Syed Naqvi, Mary F. McGuire, Jamie Buryanek, Ron J. Karni

**Affiliations:** 1 Department of Pathology and Laboratory Medicine, at UT Health McGovern Medical School, Houston, TX USA; 2 Department of Otorhinolaryngology, Head and Neck Surgery at UT Health McGovern Medical School, Houston, TX USA

**Keywords:** HPV-associated oropharyngeal carcinoma, E6/E7 in-situ hybridization, Morphoproteomics, Biomedical analytics, Biology, EZH2, Therapeutic options

## Abstract

**Background:**

Human papillomavirus (HPV) has been identified as an etiopathogenetic factor in oropharyngeal squamous cell carcinoma. The HPV *E6* and *E7* oncogenes are instrumental in promoting proliferation and blocking differentiation leading to tumorigenesis. Although surgical intervention can remove such tumors, the potential for an etiologic field effect with recurrent disease is real. A downstream effector of E7 oncoprotein, enhancer of zeste homolog 2 (EZH2), is known to promote proliferation and to pose a block in differentiation and in turn, could lead to HPV-induced malignant transformation. However, the EZH2 pathway is amenable to low toxicity therapies designed to promote differentiation to a more benign state and prevent recurrent disease by inhibiting the incorporation of HPV into the genome. This is the first study using clinical specimens to demonstrate EZH2 protein expression in oropharyngeal carcinoma (OPC).

**Methods:**

The study included eight patients with oropharyngeal carcinoma, confirmed p16INK4a- positive by immunohistochemistry (IHC). The tissue expression of E6/E7 messenger RNA (mRNA) was measured by *RNAscope*® in-situ hybridization technology. Expression of EZH2, Ki-67, and mitotic indices were assessed by morphoproteomic analysis. Biomedical analytics expanded the results with data from Ingenuity Pathway Analysis (IPA) and KEGG databases to construct a molecular network pathway for further insights.

**Results:**

Expression of *E6* and *E7* oncogenes in p16INK4a- positive oropharyngeal carcinoma was confirmed. EZH2 and its correlates, including elevated proliferation index (Ki-67) and mitotic progression were also present. Biomedical analytics validated the relationship between HPV- E6 and E7 and the expression of the EZH2 pathway.

**Conclusion:**

There is morphoproteomic and mRNA evidence of the association of p16INK4a-HPV infection with the *E6* and *E7* oncogenes and the expression of EZH2, Ki-67 and mitotic progression in oropharyngeal carcinoma. The molecular network biology was confirmed by biomedical analytics as consistent with published literature. This is significant because the biology lends itself to targeted therapeutic options using metformin, curcumin, celecoxib and sulforaphane as therapeutic strategies to prevent progression or recurrence of disease.

## Background

HPV-associated oropharyngeal carcinoma (OPC) has been reported to account for up to 60% of this subtype of head and neck cancer cases [1]. The E7 oncoprotein of HPV has been linked with the upregulation of p16INK4a protein, which serves as a surrogate marker of HPV-associated oropharyngeal carcinoma [1]. Although the prognosis of HPV-associated oropharyngeal carcinoma has been associated with a 58% reduction in mortality risk vis-à-vis the HPV-negative cases [1, 2], there is still the risk of recurrent disease and an opportunity for therapeutic intervention. Relatedly, high EZH2 expression in patients with head and neck squamous cell carcinoma was associated with advanced T stage and portended a poor survival outcome [3]. A possible connection is that E7 oncoprotein in cervical squamous cell carcinoma has been associated with the activation of EZH2 expression by HPV16 E7 at the transcriptional level [4]. However, no reports of the association of E7 in HPV-associated OPC and EZH2 pathway expression in clinical specimens from patients with OPC are currently cited in the National Library of Medicine’s MEDLINE Database (https://www.ncbi.nlm.nih.gov/pubmedhealth/).

The purpose of this report is to address gaps in the knowledge of EZH2 expression in OPC patient specimens by: 1. providing morphoproteomic and mRNA evidence of the association of p16INK4a-HPVinfection with the *E6* and *E7* oncogenes in oropharyngeal carcinoma; 2. documenting and correlating both the expression of E6 mRNA in such cases with cell cycle progression and the expression of E7 mRNA with p16INK4a, EZH2 and Ki-67 and mitotic progression; 3. confirming the biology of HPV-associated oropharyngeal carcinoma with biomedical analytics; and 4. investigating targeted therapeutic options based on biomedical analytics and preclinical data.

## Methods

### Patient population

Eight adult patients (7 males and 1 female) ranging in age from 51 to 72 years were included in this study. The anatomical locations of their biopsy-proven squamous cell carcinomas included palatine tonsil in five and tongue base in three.

### Data collection protocols

Data collection and molecular analyses were performed in accordance with the guidelines of the University of Texas McGovern Medical School Committee for the Protection of Human Subjects Institutional Review Board (IRB).

### Molecular analyses

Molecular analyses included in-situ hybridization for the expression of HPV-HR18 E6/E7 mRNA using the *RNAscope*® technology from Advanced Cell Diagnostics (https://acdbio.com/). Morphoproteomic analysis and biomedical analytics were also performed as part of the molecular analysis in our CLIA and CAP certified Consultative Proteomics Laboratory in order to define the biology of the patients’ tumors, to provide correlative expressions, and to ascertain targeted therapeutic options designed to reduce the progression or recurrence of the HPV-associated oropharyngeal carcinomas.

### In-situ hybridization

*RNAscope*® 2.5HD Red Assay was performed to evaluate expression in all 8 tissue specimens. The test assayed 18 high-risk HPV serotypes: HPV-HR18 HPV 16, 18, 26, 31, 33, 35, 39, 45, 51, 52, 53, 56, 58, 59, 66, 68, 73 and 82, E6/E7 mRNA. Hs-PPIB was used as a positive control marker for sample quality control (QC) and to evaluate RNA quality in all the tissue samples. Bacterial gene dapB was used as a negative control. Standard pretreatment assay conditions were determined to be optimal for the samples in the study set. All the samples in the study passed QC with strong PPIB expression and no/negligible dapB background. A semi-quantitative scoring system of 0-4 was utilized.

### Morphoproteomics

Morphoproteomic analysis applies bright field microscopy and immunohistochemistry directed against various protein analytes to define the biology of a neoplastic process. The analysis uncovers etiopathogenetic occurrences that might be responsible for the process development and the propensity for it to recur [5, 6]. Immunohistochemical probes were applied against the following protein analytes in unstained sections of the patients’ oropharyngeal carcinomas: Ki-67 (G1, S, G2 and M phases of the cell cycle; DakoCytomation, Carpinteria, California, lot #20001030); and enhancer of zeste homolog 2 (EZH2; Cell Signaling Technology, Inc., lot #7). The level of expression of the analytes was graded on a 0 to 3+ scale based on signal intensity indicated by a 3,3′- tetrahydrochloride (DAB) chromogenic (brown) signal, the nuclear estimation of Ki-67 and EZH2 percentages, and mitotic index based on mitotic figures/10 high power fields. The details of the morphoproteomic staining procedure have been previously described [5, 6].

### Biomedical analytics

To gain insights into HPV-associated oropharyngeal carcinoma, a standard IPA oropharyngeal pathway network (“ORO”) was constructed from key molecules associated with oropharyngeal carcinoma in the Ingenuity Knowledge Base (www.ingenuity.com). Since IPA does not include viral species, E6/E7 and their interactions associated with HPV (hsa05203) (“HPV” network) were extracted from the KEGG pathway database (http://www.genome.jp/kegg/pathway.html) and manually added to the ORO network. A “patient” pathway network was also constructed from the median patient scores and linked to ORO-HPV. From these graphs and additional data mining of the National Library of Medicine’s MEDLINE database, a single ORO-HPV network model was constructed using IPA Pathway Designer to represent the key modulation and adaptive responses in the signal transduction processes. Therapies were then linked to the ORO-HPV network model to assess potential benefits.

### Results

**Fig. 1:**
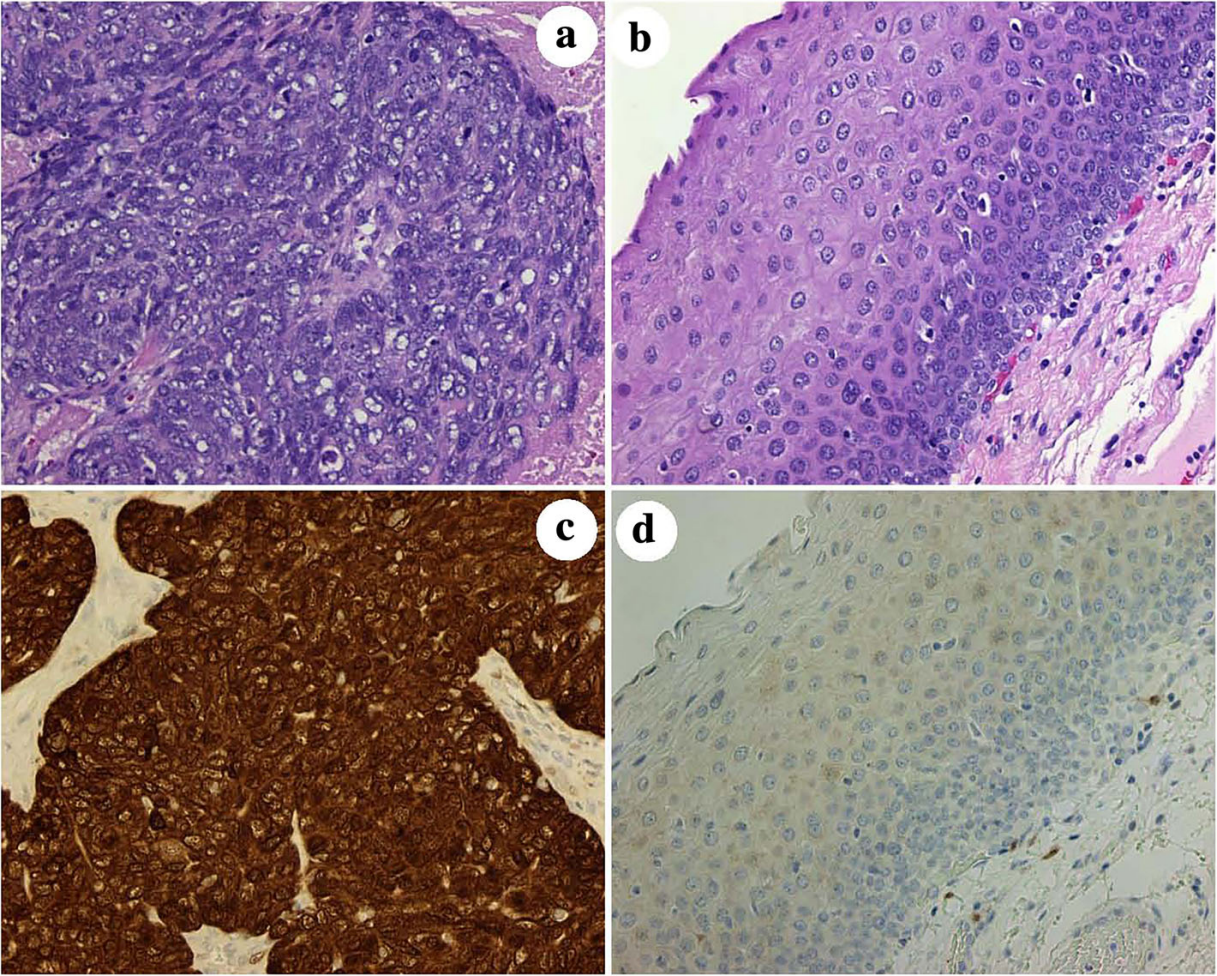
Patient 8 biopsy specimen with non-keratinizing oropharyngeal carcinoma compared with concurrent non-neoplastic mucosa. H&E and p16INK4a stained sections of non-keratininzing squamous cell carcinoma versus non-neoplastic squamous epithelium (Frames **a** and **c** and **b** and **d**, respectively; note strong DAB brown chromogenic signal for p16INK4a in oropharyngeal carcinoma [Frame **c**] versus absence of expression in non-neoplastic squamous mucosa [Frame **d**]; original magnification ×200 Frames **a**-**d**)

The IHC workup had established the expression of p16INK4a protein in all cases (Fig. 1, Table 1).

**Table 1:** Summary of median scores for percentages of Ki-67, p16INK4a, and EZH2, for mitotic indices and for E6/E7 mRNA by individual patient with oropharyngeal carcinoma

Patient	1	2	3	4	5	6	7	8	Median
Ki-67 (MKI67)	80	85	90	60	70	70	60	60	70
Mitotic Index	105	44	36	40	16	55	31	96	42
p16 INK4a (CDKN2A)	100	100	100	100	100	100	100	100	100
EZH2	90	90	90	90	90	90	80	80	90
E6/E7 mRNA	100	100	100	100	50	75	100	100	100

### In-situ hybridization

**Fig. 2:**
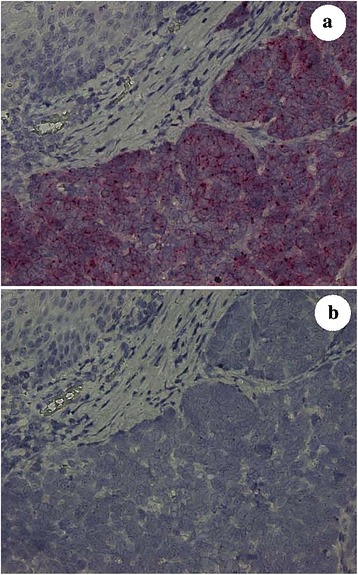
Patient 8 biopsy specimen with non-keratinizing oropharyngeal carcinoma compared with adjacent non-neoplastic mucosa. Red *RNAscope®* 2.5 HD in-situ hybridization (ISH) assay for HPV-HR18 E6/E7 mRNA performed on the non-keratinizing squamous cell carcinoma revealed strong red chromogenic cytoplasmic expression (4+ semi-quantitative score, see Table 2) in the tumor (lower right and middle, Frame **a**) and no expression in the adjacent non-neoplastic (upper left, Frame **a**). Contrast with the dapβ negative control in Frame **b**. (original magnification ×200 for Frames a and **b**)

HPV-HR18 E6/E7 was detected at high levels across most samples (score 4) with the exception of patient specimens 5 and 6 that scored at 2 and 3, respectively (see Table 2 and Fig. 2).

**Table 2:** HPV-HR18-E6/E7 mRNA semi-quantitative scoring data by individual patient with oropharnygeal carcinoma

Patient Specimen ID	Hs-PPIB Score (positive control)	dapβ Score (negative control)	QC Pass/Fail	HPV-HR18 E6/E7 mRNA Score
1	4	0	Pass	4
2	4	0	Pass	4
3	4	0	Pass	4
4	4	0	Pass	4
5	3	0	Pass	2
6	3	0	Pass	3
7	4	0	Pass	4
8	4	0	Pass	4

### Morphoproteomics

**Fig. 3:**
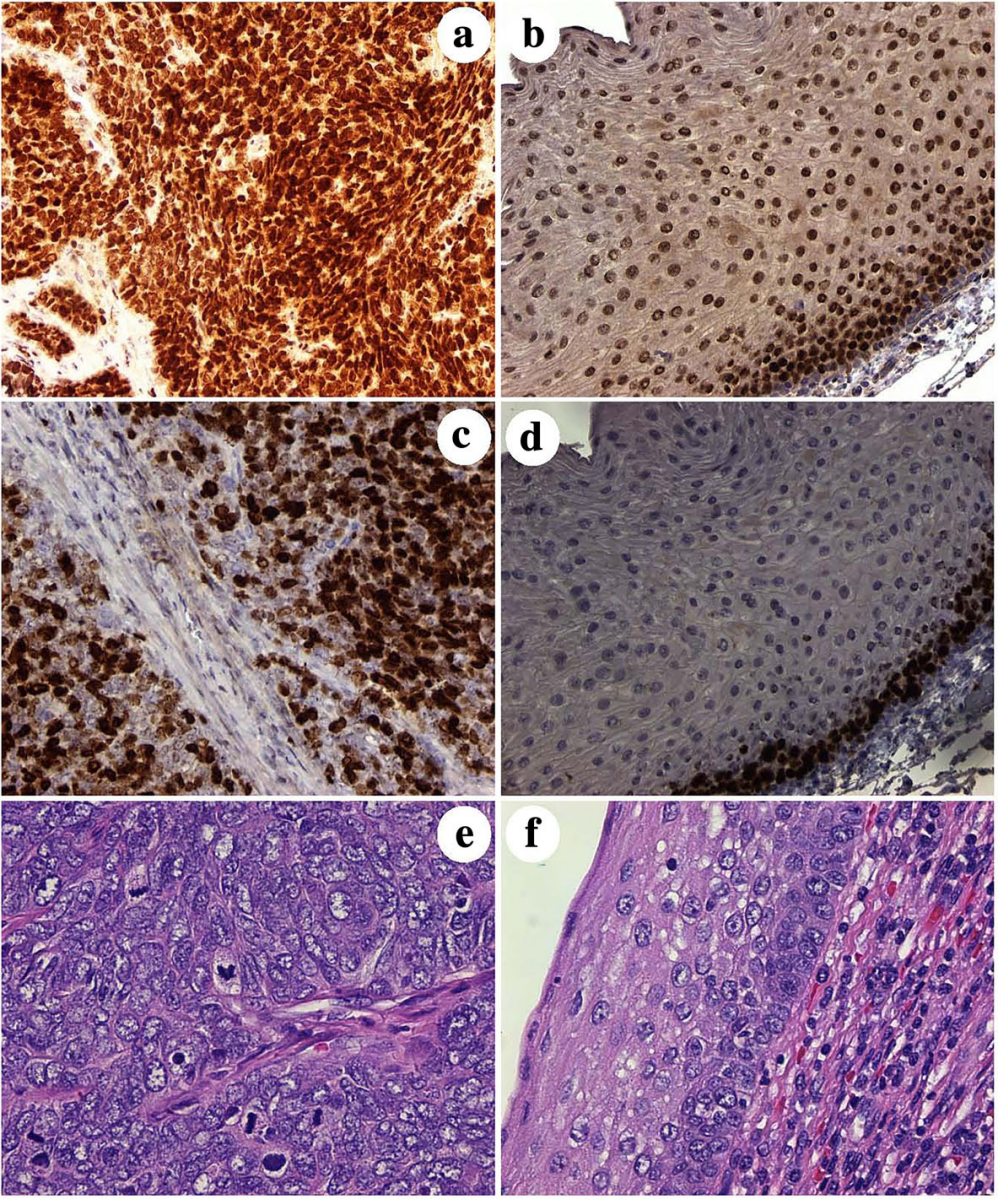
Patient 8 biopsy specimen with non-keratinizing oropharyngeal carcinoma compared with concurrent non-neoplastic mucosa. Enhancer of zeste homolog 2 (EZH2) and Ki-67 (G1, S, G2 and M phases of the cell cycle) show strong (3+ on a scale of 0-3+) nuclear expression in a majority of the non-keratinizing squamous cell carcinoma (NKSCC) versus similar expression primarily limited to the basal and suprabasal cells of the non-neoplastic squamous mucosa (Frames **a** and **c** versus **b** and **d**, respectively). Mitotic progression in the corresponding H&E coincides with the EZH2 and Ki-67 expression with multiple mitotic figures evident in the NKSCC (Frame **e**) with no mitotic figures in the adjacent non-neoplastic squamous mucosa (Frame **f**). (DAB brown chromogenic signal for frames **a**-**d**; original magnifications ×200 for frames **a**-**d** and ×400 for Frames **e** and **f**)

Nuclear expressions of EZH2 (enhancer of zeste homolog 2) and Ki-67 (G1, S, G2 and M phases of the cell cycle) and mitotic indices (mitotic figures/10 high power fields) by visual estimation on each case revealed a median of 90% and 70% for EZH2 and Ki-67, respectively and a range of 16 to 105 for the mitotic indices (Table 1 and Fig. 3, frames a and b, c and d, and e and f, respectively for EZH2, Ki-67 expression, and mitotic progression).

### Biomedical analytics

**Fig. 4:**
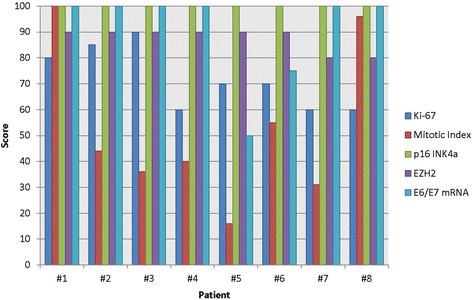
Bar chart of normalized (median) score by patient of the comparative analytes for Ki67, mitotic index, p16INK4a,EZH2 and E6/E7 mRNA. Comparison of normalized scores. Note that patient #5 has the lowest E6/E7 score (assessed as moderate, 2+), and also the lowest mitotic index (see Tables 1 and 2)

In order to provide a visual comparison, biomedical analytics generated a normalized score by patient of the comparative analytes for Ki67, mitotic index, p16INK4a, EZH2 and E6/E7 mRNA. This is illustrated in the bar chart (Fig. 4).

#### ORO-HPV network model

There were extensive crossover interactions between the HPV pathway and the ORO pathway. Three molecules of interest - EZH2, MKI67 (Ki67) and CDKN2A (p16 INK4a) were present – and affected – in the combined pathway network (not shown.) This combined ORO-HPV network model contained 1086 molecular interactions, or links, in the 3 linked pathways of ORO, HPV, and median patient. Of the 1086 links, 513 bridged between the ORO network and the HPV network. For the patient, the level of CDKN2A affected more molecules associated with HPV than with ORO. In return, more molecules from HPV than ORO affected the patient network, with TP53 and RB1 being the major influencers. 174 molecules from ORO affected HPV and patients; whereas 224 molecules from HPV affected ORO and patient*.*

#### Therapeutic interactions

**Fig. 5:**
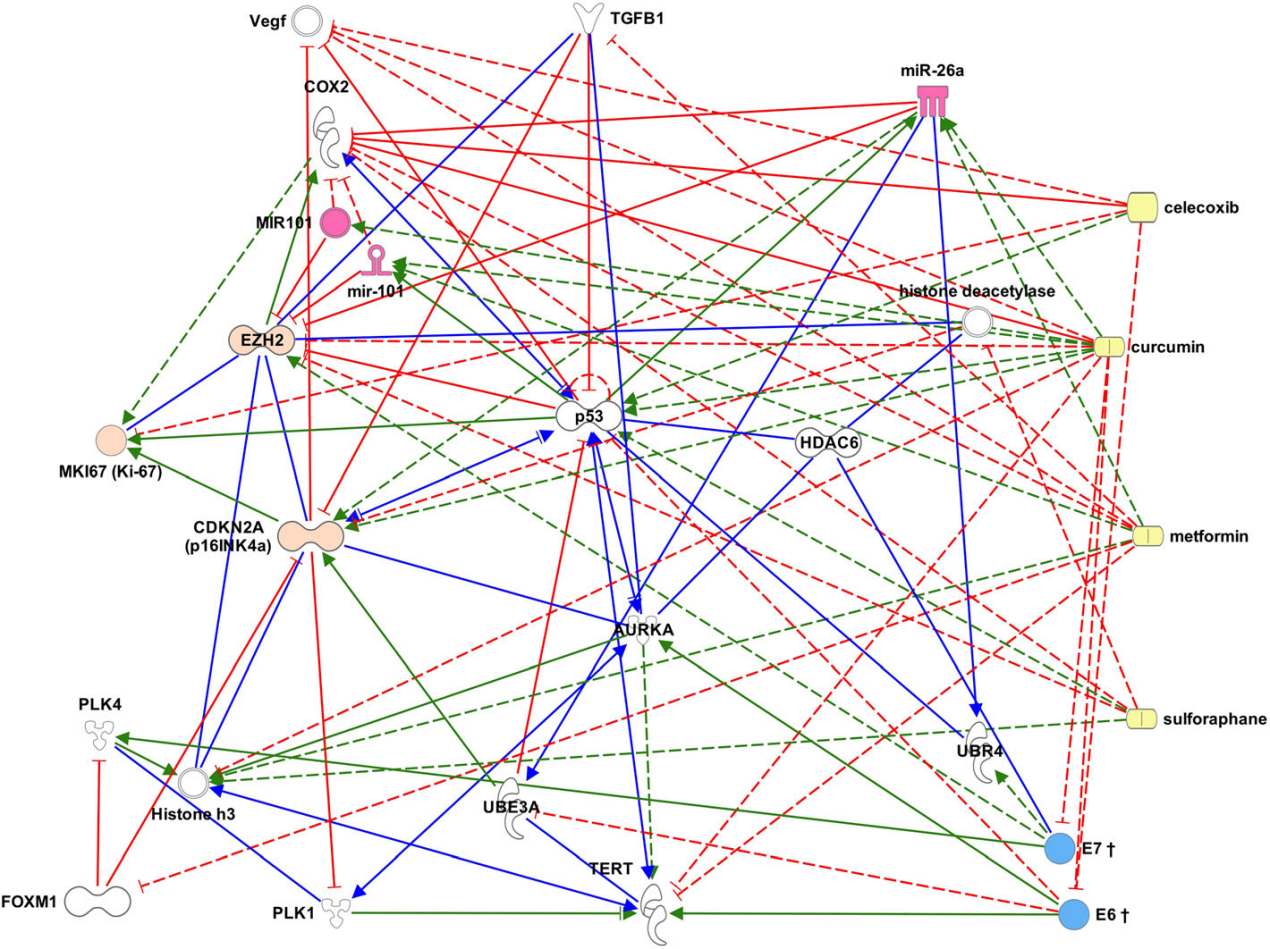
Potential therapies for ORO-HPV combined pathway. Celecoxib, curcumin, metformin, sulforaphane (*right, yellow*). Scored molecules: CDKN2A, EZH2, MKI67 (*left, light orange*). E6, E7 (*lower right, blue, viral* †). microRNAs: miR-26a, MIR101, miR-101 (*top, pink*)

The complex ORO-HPV network model was edited to focus on the key molecules: E6/E7 mRNA, EZH2, Ki-67 (MK167) plus related interactions from the National Library of Medicine’s MEDLINE Database. The potential efficacy of sulforaphane and the metformin and curcumin therapies – the latter in part through the upregulation of microRNAs – were graphically demonstrated (Fig. 5). EZH2 was identified as a possible therapeutic target. It can be seen from the networks that EZH2 (Fig. 5, *upper left*) is a key network point that is activated by E6/E7. Mir-26A can be seen in the upper right hand corner of Fig. 5; it needs to be upregulated to decrease EZH2.

## Discussion

This report provides morphoproteomic and mRNA evidence of the association of p16INK4a-HPVinfection with the *E6* and *E7* oncogenes in oropharyngeal carcinoma. The E6 oncoprotein has been shown to bind with and promote the degradation of wild type p53 by activating the ubiquitin ligase E6AP [1, 7], and thereby, potentially to facilitate cell cycle progression [1, 8, 9]. Similarly, the E7 oncoprotein via the upregulation of EZH2 should promote cell cycle progression [1, 4]. Morphoproteomics and biomedical analytics provide correlates in our cases in the form of Ki-67 (G1, S, G2 and M phases) with mitotic progression. The seemingly paradoxical but ineffective increase in p16INK4a, a cyclin-dependent kinase inhibitor [10], coincides with E7 expression in our cases of oropharyngeal carcinoma [1]. In addition to its association with cell cycle progression in our study, enhancer of Zeste homolog 2 (EZH2) – a histone methyltransferase – is potentially tumorigenic by virtue of the fact that it methylates and inactivates tumor suppressor genes and contributes to a state of proliferation and dedifferentiation in tumors and in facilitating their migratory potential [11–16]. This could account, in part, for the non-keratinizing/poorly keratinizing component of HPV-associated squamous cell carcinomas. The recent manuscript by Idris et al. [17] showed that inhibition of EZH2 has anti-tumorigenic effects on oropharyngeal squamous cell carcinoma (OPSCC) cells in culture that is more pronounced in HPV-positive cell lines. This preclinical evidence plus the results of our study support the applicability of our approach for human patients.

Biomedical analytics confirmed the correlations and the interactive biology of E6/E7 mRNA-associated oropharyngeal carcinoma with the morphoproteomic findings in our study and illustrated the potential targeted therapies of metformin, curcumin, celecoxib and sulforaphane.

Rationale for the application of these agents against HPV-associated oropharyngeal carcinoma are provided as follows: pharmacogenomic upregulation of microRNAs, miR-26a and miR-101, by metformin and a curcumin analog in preclinical studies correspondingly decreased the expression of EZH2 [18, 19] and metformin delays cell cycle progression [20]; curcumin has anti-tumoral activity against HPV-associated cells by selectively inhibiting the expression of viral oncogenes *E6* and *E7* [21, 22]; celecoxib in HPV-18-infected cervical cancer cells has been shown to restore p53 by suppressing viral oncoprotein E6 expression by down-modulating COX-2 and retrieving p53 from COX-2 association [23]; and sulforaphane suppresses EZH2 expression in skin cancer cells via a proteasome-dependent mechanism [24]. Lindsay et al. recently underscored the opportunities for novel therapeutic targets, such as EZH2, in OPC [25]. Although our pilot study was limited by a small patient population, the results are encouraging. We are in the process of developing a specific clinical protocol for patients with a confirmatory biopsy of HPV -oropharyngeal carcinoma (p16INK4a+/EZH2+) with high Ki-67 expression and mitotic progression. The protocol would incorporate the listed therapeutic agents in a combinatorial fashion and be applied following a confirmatory biopsy of HPV-oropharyngeal carcinoma (p16INK4a+/EZH2+ with high Ki-67 expression and mitotic progression) prior to the implementation of standard tumor board-recommended therapy for the individual patient.

## Conclusions

Our study showed that p16INK4a-HPV infection is associated with the *E6* and *E7* oncogenes and with the expression of EZH2, Ki-67, and mitotic progression in oropharyngeal carcinoma. This biology lends itself to targeted therapeutic options using metformin, curcumin, celecoxib and sulforaphane as therapeutic strategies to prevent progression or recurrence of disease.

## Abbreviations

CAP: College of American Pathologists
CLIA: Clinical Laboratory Improvement Amendments
DAB: 3,3'-Diaminobenzidine
EZH2: Enhancer of zeste homolog 2
H&E: Hematoxylin and eosin stain
HPV: Human papillomavirus
IHC: Immunohistochemistry
IPA: Ingenuity Pathway Analysis
IRB: Institutional Review Board
KEGG: Kyoto encyclopedia of genes and genomes
Ki-67/MKI67: Proliferation marker protein Ki-67
mRNA: messenger ribonucleic acid
p16INK4a/CDKN2A: Cyclin-dependent kinase inhibitor 2A
QC: Quality control
RB1: Retinoblastoma-associated protein
RNA: Ribonucleic acid.
